# Pharmacologic Thromboprophylaxis in Medical Inpatients: A Systematic Review and Network Meta-Analysis

**DOI:** 10.1001/jamanetworkopen.2026.11449

**Published:** 2026-05-15

**Authors:** Christophe Marti, Marc Righini, Grégoire Le Gal, Laura Girardi, Helia Robert-Ebadi, Christophe Combescure, Jean-Luc Reny, Marc Blondon

**Affiliations:** 1 Division of General Internal Medicine, University Hospitals of Geneva, Geneva, Switzerland; 2 Faculty of Medicine, University of Geneva, Geneva, Switzerland; 3 Division of Angiology and Haemostasis, University Hospitals of Geneva, Geneva, Switzerland; 4 Department of Medicine, Ottawa Hospital Research Institute, University of Ottawa, Ottawa, Ontario, Canada; 5 Research Center on Thromboembolic Diseases and Antithrombotic Treatment, Department of Medicine and Surgery, University of Insubria, Varese, Italy; 6 Division of Clinical Epidemiology, University Hospitals of Geneva, Geneva, Switzerland

## Abstract

**Importance:**

Pharmacologic thromboprophylaxis is widely used for medical inpatients, but its contemporary benefits remain uncertain for clinically relevant venous thromboembolism (VTE).

**Objective:**

To evaluate and compare the benefits and risks associated with currently approved in-hospital pharmacologic thromboprophylaxis regimens to prevent symptomatic and clinically relevant VTE and bleeding in acutely ill medical inpatients.

**Data sources:**

MEDLINE, Embase, Web of Science, and the Cochrane CENTRAL databases were searched until January 31, 2026. Reference lists from retrieved articles and reference literature were also examined.

**Study Selection:**

Randomized clinical trials that involved adult patients hospitalized for an acute medical illness and compared pharmacologic thromboprophylaxis regimens (low-molecular-weight heparin [LMWH], unfractionated heparin [UHF], and direct oral anticoagulants [DOACs]) with no treatment or placebo were selected.

**Data Extraction and Synthesis:**

Dual independent screening, data extraction, and risk-of-bias assessment were performed. Relative risks (RRs) were pooled using network meta-analyses with random effects. The PRISMA reporting guideline was used.

**Main Outcomes and Measures:**

The effectiveness outcomes were the risk of confirmed symptomatic VTE, clinically relevant VTE, and any VTE. Safety outcomes were mortality, major bleeding, and clinically relevant nonmajor bleeding. Pairwise and network meta-analyses were computed.

**Results:**

Among the 22 studies (involving 43 840 patients) included, the 90-day pooled risk of symptomatic VTE was 1.7% (95% CI, 0.6%-4.4%) in the no-treatment group. Compared with no treatment, LMWH was associated with reduced symptomatic VTE risk (RR, 0.68; 95% CI, 0.49-0.94). Point estimates for DOACs (RR, 0.69; 95% CI, 0.36-1.31) and UFH (RR, 0.75; 95% CI, 0.40-1.40) were less than 1, although the results were not statistically significant. LMWH (RR, 0.57; 95% CI, 0.43-0.74), DOACs (RR, 0.58; 95% CI, 0.41-0.82), and UFH (RR, 0.66; 95% CI, 0.45-0.97) were associated with reduced risk of clinically relevant VTE. Using a theoretical conversion rate of asymptomatic into symptomatic VTE, both LMWH and DOACs were associated with decreased risk of symptomatic VTE (RR, 0.63 [95% CI, 0.48-0.83] and 0.66 [95% CI, 0.44-0.99]). DOACs (RR, 2.62; 95% CI, 1.25-5.49) and UFH (RR, 2.33; 95% CI, 1.13-4.79) were associated with an increased risk of major bleeding, compared with no treatment, while LMWH (RR, 1.23; 95% CI, 0.81-1.85) had no association. Mortality was unaffected by the treatment regimens.

**Conclusions and Relevance:**

In this systematic review and network meta-analysis, DOACs, LMWH, and UFH were associated with reduced clinically relevant VTE in medical inpatients; DOACs and UFH had higher risks of major bleeding than LMWH. Proper selection of patients is critical given the overall low absolute risk of VTE.

## Key Points

**Question:**

What are the risks and benefits associated with in-hospital thromboprophylaxis in medical inpatients?

**Findings:**

In this systematic review and network meta-analysis of 22 randomized clinical trials, pharmacologic thromboprophylaxis was associated with relative risk reduction of symptomatic and clinically relevant venous thromboembolism. Low-molecular-weight heparin was associated with a lower risk of major bleeding.

**Meaning:**

The finding that pharmacologic thromboprophylaxis reduced clinically relevant venous thromboembolism suggests that proper selection of patients is critical given the overall low absolute risk of venous thromboembolism.

## Teaser

This systematic review and network meta-analysis examines the risk of venous thromboembolism, mortality, and bleeding associated with low-molecular-weight heparin, unfractionated heparin, and direct oral anticoagulants among acutely ill hospitalized patients.

## Introduction

Patients hospitalized for acute medical illness face an increased risk of venous thromboembolism (VTE).^1^ Medical inpatients are particularly exposed to several thrombogenic risk factors, such as acute infection, cancer, indwelling catheters, and reduced mobility.

Given the burden of hospital-associated VTE, several landmark studies have evaluated the effectiveness of pharmacologic regimens, including unfractionated heparin (UFH) and low-molecular-weight heparin (LMWH), in preventing VTE outcomes.^2,3,4,5,6,7^ The findings have led to a broad use of in-hospital thromboprophylaxis.

However, recommendations for the use of thromboprophylaxis in most admitted patients have been challenged for several reasons.^8^ First, the primary composite VTE outcomes in many trials included radiologically detected clinically irrelevant events, such as asymptomatic distal deep vein thrombosis (DVT). Whether the effectiveness of thromboprophylaxis is similar for patient-relevant outcomes is uncertain. In a previous meta-analysis including patients with stroke and patients in intensive care units (ICUs), Eck et al^9^ did not show a significant reduction in symptomatic VTE for patients treated with UFH, pentasaccharides, and direct oral anticoagulants (DOACs), compared with placebo. Intermediate-dose LMWH was the only comparator associated with a substantial decrease in symptomatic VTE. Second, a bias may arise from simply excluding asymptomatic VTE events diagnosed through systematic screening, as some of these events could evolve if untreated to symptomatic and/or clinically relevant events. Third, a large international clinical trial reported a nonsignificant reduction of symptomatic VTE at 30 and 90 days.^10^ How these results modify the global evidence on pharmacologic thromboprophylaxis in medical inpatients is important to assess.

Given these contrasting observations and the magnitude of resources allocated to hospital VTE prevention, we performed a systematic review and network meta-analysis. Our aim was to evaluate and compare the benefits and risks of currently approved in-hospital pharmacologic thromboprophylaxis regimens to prevent symptomatic and clinically relevant VTE and bleeding in acutely ill medical inpatients.

## Methods

### Protocol and Registration

The search strategy, study selection, data extraction, and data analysis were performed according to a predefined protocol (PROSPERO identifier: CRD42024505871). We followed the Preferred Reporting Items for Systematic Reviews and Meta-analyses (PRISMA) reporting guideline.

### Eligibility Criteria

Randomized clinical trials (RCTs) of adult patients (≥18 years) hospitalized for an acute medical (nonsurgical) illness were eligible. We excluded studies that selectively included specific subgroups of inpatients with stroke or myocardial infarction, active cancer, or COVID-19 acute infection or in the ICU, as these populations have different baseline thrombotic or bleeding risks and co-medications.^11,12^

To provide findings relevant to clinical practice, we included RCTs evaluating currently approved regimens for in-hospital thromboprophylaxis, divided into 3 groups: LMWH, UFH, and DOACs. Details regarding eligibility criteria, study selection, and quality assessment are provided in eTable 1 in Supplement 1.

### Data Sources and Searches

Two authors (M.B. and C.M.) systematically searched MEDLINE, Embase, Web of Science, and the Cochrane CENTRAL (Central Register of Controlled Trials) databases until January 31, 2026, using a comprehensive search strategy. The search strategy is described in eTable 2 in Supplement 1. We also examined reference lists from retrieved articles and reference literature.

### Study Selection and Data Extraction

Two authors (M.B. and C.M.) independently evaluated studies for inclusion using a shared blinded software (Covidence; The Cochrane Collaboration). Details are provided in eTable 1 in Supplement 1.

We a priori defined 2 main VTE outcomes of clinical relevance to patients. The primary outcome was the risk of confirmed symptomatic VTE, including fatal pulmonary embolism (PE), symptomatic PE, and/or symptomatic proximal or distal lower limb DVT, according to adjudication within each study. The secondary outcome of interest was clinically relevant VTE, defined as symptomatic VTE and asymptomatic proximal lower limb DVT. Asymptomatic proximal DVT is a relevant clinical outcome because it is associated with higher risk of PE and increased mortality.^13^ A tertiary outcome was any VTE, including symptomatic or asymptomatic PE, proximal DVT, and/or distal lower limb DVT.

We considered confirmed diagnostic tests for PE: lung scintigraphy, computed tomography pulmonary angiography, and pulmonary angiography. For DVT, we considered leg phlebography, lower limbs vein compression ultrasonography, and ^125^I-labeled fibrinogen scanning. Safety outcomes included major bleeding, using the definition provided by individual studies; clinically relevant nonmajor bleeding (CRNMB); and all-cause mortality (eTable 1 in Supplement 1).^14^

We primarily evaluated outcomes at 3 months or 90 days (or closest available data) because the preventive effectiveness of hospital thromboprophylaxis might extend longer than the treatment period itself.^10^ A sensitivity analysis was conducted using the 28-day outcomes.

### Consideration for VTE Screening in Trials

Several RCTs used systematic screening for asymptomatic proximal DVT or proximal or distal DVT. In such trials, participants with asymptomatic DVT were usually treated with therapeutic anticoagulation, thus artificially reducing the risk of developing symptomatic DVT or PE. Simply excluding these asymptomatic events might lead to an underestimated benefit of thromboprophylaxis to prevent symptomatic VTE, especially if these events occur predominantly in the control arm. To consider this potential bias, we a priori planned a sensitivity analysis to estimate the number of asymptomatic DVT diagnosed by screening that might have resulted in symptomatic events had screening not been performed. Based on previous evidence,^15,16,17^ we used conversion ratios of 7.9% of screened asymptomatic distal DVT converted into symptomatic VTE and 60% of screened asymptomatic proximal DVT converted into symptomatic VTE (eTable 3 in Supplement 1). We added these hypothetical events to symptomatic VTE in a sensitivity analysis and tested lower and higher conversion rates (3% and 40%; 15% and 80%).

### Quality Assessment

We used the quality criteria of the Cochrane Collaboration risk-of-bias assessment tool,^18^ implemented in Covidence at the time of study extraction. Details of this assessment are provided in eTable 1 in Supplement 1.

### Statistical Analysis

We computed pooled risks for the different treatment arms (LMWH, UFH, and DOACs) using random effect models. Relative risks (RRs) for comparison between treatment arms were pooled across RCTs using network meta-analyses with random effects.^19^ Estimates were obtained by using a frequentist method with the package Netmeta for R, version 3.5.0 (R Project for Statistical Computing). To further assess the inconsistency of the network, we also reported estimates for direct comparisons for each pair of compared treatments.

For the primary outcome, we performed sensitivity analyses to evaluate the strength of the pooled RRs. We added the screening conversion rate, restricted to 28-day outcomes, applied the leave-one-out principle, and excluded studies using ^125^I-labeled fibrinogen scanning.

The 2-sided statistical significance level was set at *P* = .05 for all analyses, except for the heterogeneity and inconsistency tests with an α = .10.^20^ To determine treatment rankings for effectiveness and safety outcomes, we derived P-scores from the network meta-analysis. The P-score can be interpreted as the mean extent of certainty that treatment is better than another treatment, averaged over all competing treatments, and can be seen as the frequentist equivalent of the Surface Under the Cumulative Ranking value.^21^

To facilitate the interpretation of pooled RRs, we derived the effect sizes from absolute risk differences for the main effectiveness and safety outcomes by applying RRs to the pooled baseline risk in the control arm (ie, no treatment). We evaluated the strength of evidence using the Grading of Recommendations Assessment, Development, and Evaluation (GRADE) criteria for each outcome for LMWH.^22^ We evaluated the presence of publication bias by visual evaluation of funnel plots and by the trim-and-fill method for outcomes including at least 5 studies.^23^

## Results

### Study Selection and Characteristics

The search retrieved 2906 references (eFigure 1 in Supplement 1). We evaluated 65 full texts, of which 22 fulfilled the inclusion criteria (interrater reliability: 0.82). These 22 RCTs included 43 840 medical inpatients. The characteristics of these trials are described in Table 1 and eTable 4 in Supplement 1. Of the 22 studies, 4 (18.2%) compared UFH with no treatment,^2,3,24,27^ 9 (40.9%) compared UFH with LMWH,^25,26,28,29,30,33,34,35,38^ 7 (31.8%) compared LMWH with no treatment,^5,6,7,10,17,31,32^ and 2 (9.1%) compared DOACs with LMWH.^36,37^ The diagram of the network is provided in eFigure 2 in Supplement 1.

**Table 1.** Characteristics of the Included Randomized Clinical Trials^a^

Source	Design	Inclusion criteria	Treatment regimen		Duration of intervention	Duration of follow-up	Available outcomes for analysis	VTE screening modality	No. randomized; No. for primary effectiveness analysis
			Intervention	Control					
Gallus et al,^3^ 1973	Open label	Inpatients with suspected MI or HF	UFH 5000 IU 3 times per day	No treatment	Until mobility (mean, 10.6 d; range, 5-21 d)	Until mobility or discharge	VTE	^125^I-labeled fibrinogen scan every 1-2 d until mobility or discharge	78; 78
Belch et al,^2^ 1981	Open label	40-80 y, Inpatients with HF and/or pulmonary infection	UFH 5000 IU 3 times per day	No treatment	Until mobility (mean, 8.5 d)	Until discharge	VTE	^125^I-labeled fibrinogen scan every 2 d until 14 d or discharge	100; 100
Ibarra-Pérez et al,^24^ 1988	Open label	≥40 y, Inpatients with pulmonary disease	UFH 5000 IU twice per day	Mechanical thromboprophylaxis (GCS and elastic bandages)	Until mobility (mean, 7-9 d)	Until mobility	VTE	^125^I-labeled fibrinogen scan, CUS, and plethysmography every day	146; 111 (excluding the aspirin group)
Aquino et al,^25^ 1990	Open label	>70 y, Geriatric inpatients who might benefit from prophylactic heparin, >45 kg, without DVT at baseline	Nadroparin 0.3 mL (2850 IU) once per day^b^	UFH 5000-7500 IU twice per day	NA	NA	VTE, MB, mortality	CUS	99; 99
Forette and Wolmark,^26^ 1995	Open label	≥70 y, Hospitalization presumed for ≥4 wk, with recently decreased mobility	Nadroparin 3075 IU (0.3 mL) once per day	UFH 5000 IU 3 times per day or 7500 IU 3 times per day (≥70 kg)	28 d	28 d	VTE, MB, mortality	Whole-leg CUS if early study withdrawal or at 28 d	295; 295
Gärdlund,^27^ 1996	Open label	>55 y, Inpatients with infectious disease and immobility	UFH 5000 IU twice per day	No treatment	Until discharge (mean, 8.2 d)	21 d After hospital discharge (maximum: 60 d)	VTE, mortality	None	11693; 383 (necropsies)
Lechler et al,^28^ 1996	Double blind	>18 y, Inpatients with expected immobility for 7 d and 1 additional risk factor (>60 y; malignant neoplasm; obesity; previous VTE; cardiac failure; limb paresis, hemiplegia, or paraplegia; severe infection)	Enoxaparin 40 mg once per day	UFH 5000 IU 3 times per day	7 d	7 d	VTE, MB, mortality	CUS at study entry and at 7 d	959; 885
Harenberg et al,^29^ 1996	Double blind	50-80 y, Inpatients with expected bed rest of ≥10 d and 1 additional risk factor (excess weight, varicosis, previous VTE, estrogens and platelets >450 G/L, previous AT event)	Nadroparin 36 mg once per day (~3400 IU)	UFH 5000 IU 3 times per day	10 d or Until discharge	Until hospital discharge	VTE, MB, mortality	Proximal CUS on day 1 and between day 8 and 11	1968; 1590
Samama et al,^6^ 1999	Double blind	>40 y, Inpatients with HF or respiratory failure, infection, lumbar pain, arthritis, IBD with another risk factor	Enoxaparin 40 mg once per day (enoxaparin 20 mg; arm excluded)	Placebo	6-14 d (Median, 7 d)	83-110 d	VTE, MB, mortality	Venography or CUS at 6-14 d	738; 579
Kleber et al,^30^ 2003	Open label	≥18 y, Inpatients with severe respiratory disease or HF, with bed rest >two-thirds of the d	Enoxaparin 40 mg once per day	UFH 5000 IU 3 times per day	Mean (SD), 10 (2) d	~10 d	VTE, MB, mortality	Serial fibrin or D-dimer measurements after 2 and 5 d, and 1 d prior to end of treatment; venography if positive result	668; 451
Leizorovicz et al,^4^ 2004	Double blind	≥40 y, Inpatients with acute CHF or respiratory failure, infection, rheumatologic disorder, or IBD with another risk factor	Dalteparin 5000 IU once per day	Placebo	14 d or Until discharge	90 d	VTE, MB, mortality	Proximal CUS after 21-24 d	3706; 2991
Mahé et al,^31^ 2005	Double blind	>40 y, Inpatients with immobilization	Nadroparin 0.3 mL (2850 IU) once per day^b^	Placebo	21 d or Until discharge	21 d	MB, mortality	None	1474; 1474
Lederle et al,^32^ 2006	Double blind	≥60 y, Inpatients with planned stay ≥3 d	Enoxaparin 40 mg once per day	Placebo	Until discharge or 90 d	90 d	VTE, MB, mortality	None	280; 280
Cohen et al,^5^ 2006	Double blind	≥60 y, Inpatients with CHF, acute respiratory illness with chronic lung disease, acute infection, or inflammatory disorder	Fondaparinux 2.5 mg once per day	Placebo	6-14 d (Median [IQR], 7 [1.15 for fondaparinux; 1-13 for placebo] d)	32 d	VTE, MB, mortality	Venography at 6-15 d	849; 644
NCT00445328,^33^ 2009	Open label	≥18 y, Inpatients with CHF, respiratory failure, acute infection, acute rheumatologic disorder, acute IBD, or acute sciatica with at least 1 extra risk factor	Dalteparin 5000 IU once per day	UFH 5000 IU twice per day	6-14 d	21 d	VTE, MB, mortality	Probable screening for asymptomatic proximal DVT	84; 72
Riess et al,^34^ 2010	Double blind	≥70 y, Inpatients with decreased mobility for ≥4 d	Certoparin 3000 IU once per day	UFH 5000 IU 3 times per day	8-20 d (Mean [SD], 9.1 [3.4 for certoparin; 3.3 for UFH] d)	90 d	VTE, MB, mortality	Whole-leg CUS at end of treatment	3244; 2743
Schellong et al,^35^ 2010	Open label	>40 y, Inpatients	Certoparin 3000 IU once per day	UFH 7500 IU 3 times per day	10 d (Mean [SD], 8.5 [2.1] d)	90 d	VTE, MB	Whole-leg CUS at 10 d	342; 203
Kakkar et al,^7^ 2011	Double blind	≥40 y, Inpatients with HF, active cancer, or severe infection + chronic pulmonary disease, obesity, history of prior VTE, or ≥60 y	Enoxaparin 40 mg once per day + GCS	Placebo + GCS	6-14 d (Median, 9 d)	90 d	VTE, MB, mortality	None	8323; 8307
Goldhaber et al,^36^ 2011	Double blind	≥40 y, Inpatients with HF or respiratory failure, infection, acute rheumatic disorder, or IBD with at least another risk factor	Apixaban 2.5 mg twice per day	Enoxaparin 40 mg once per day	30 d (Apixaban); minimum hospital stay or 6 d (mean [SD], 7.3 [4.0] d), (enoxaparin)	90 d (only 7.3 d in LMWH phase for this analysis)	VTE, MB	Proximal CUS at end of LMWH treatment and at 30 d	6528; 4973
Cohen et al,^37^ 2013	Double blind	≥40 y, Inpatients with HF, active cancer, acute stroke, or acute infection or respiratory failure with another risk factor	Rivaroxaban 10 mg once per day	Enoxaparin 40 mg once per day	35 d (Rivaroxaban) 6-14 d (enoxaparin)	35 d (only 10 d in LMWH phase for this analysis)	VTE, MB, mortality	Proximal CUS at end of LMWH treatment (6-14 d)	8101; 5931
Ishi et al,^38^ 2013	Double blind	Inpatients with high VTE risk according to a score, with stay of ≥3 d in ICU, or in nonambulatory condition in intermediary care	Enoxaparin 40 mg once per day	UFH 5000 IU twice per day	Until ambulant and ready for discharge	Until discharge	VTE, MB	CUS at end of treatment	92; 92
Mottier et al,^10^ 2023	Double blind	≥70 y, Inpatients with life expectancy ≥3 mo and hospital stay ≥4 d	Enoxaparin 40 mg once per day	Placebo	6-14 d	90 d	VTE, MB, mortality	None	2557; 2541

Abbreviations: AT, arterial thrombotic; CHF, congestive heart failure; CUS, compression ultrasonography; DVT, deep vein thrombosis; GCS, graduated compression stockings; HF, heart failure; IBD, inflammatory bowel disease; ICU, intensive care unit; LMWH, low-molecular-weight heparin; MI, myocardial infarction; NA, not available; UFH, unfractionated heparin; VTE, venous thromboembolism.

^a^ More information is available in eTable 4 in Supplement1.

^b^ Discordant information on the nadroparin dose, but most likely 0.3 mL/2850 IU.

### Study Quality and Risk of Bias

The risk of bias in individual studies is provided in eTable 5 in Supplement 1. Randomization and allocation concealment methods were unclear in older trials (9 of 22 [40.9%]).^2,6,17,24,25,27,28,29,33^ Most studies (12 [54.6%]) were double blind,^3,4,5,6,7,10,28,29,31,34,36,37^ and incomplete outcome data were common (12 [54.6%]).^3,5,6,17,24,29,30,33,34,35,36,37^

### Clinical Outcomes

#### Symptomatic VTE

Fourteen studies (involving 34 551 patients) reported on symptomatic VTE.^4,5,6,7,10,25,26,30,32,33,34,35,36,37^ The pooled 90-day risk of symptomatic VTE was 1.7% (95% CI, 0.6%-4.4%) in the no-treatment group.

**Figure 1.**
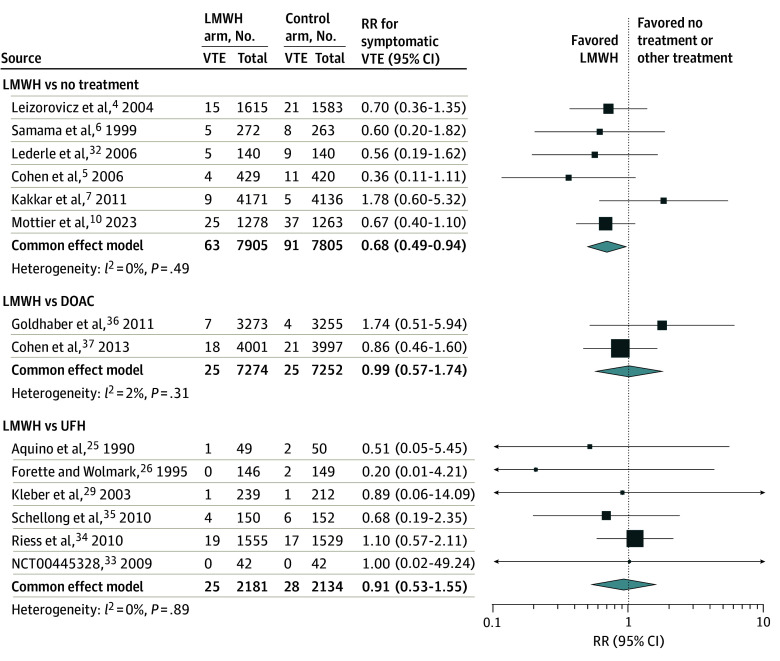
Forest Plot of Direct Meta-Analysis Comparing Treatment Regimens for the 90-d Risk of Symptomatic Venous Thromboembolism (VTE) Error bars represent 95% CIs. DOAC indicates direct oral anticoagulants; LMWH, low-molecular-weight heparin; RR, relative risk; and UFH, unfractionated heparin.

**Figure 2.**
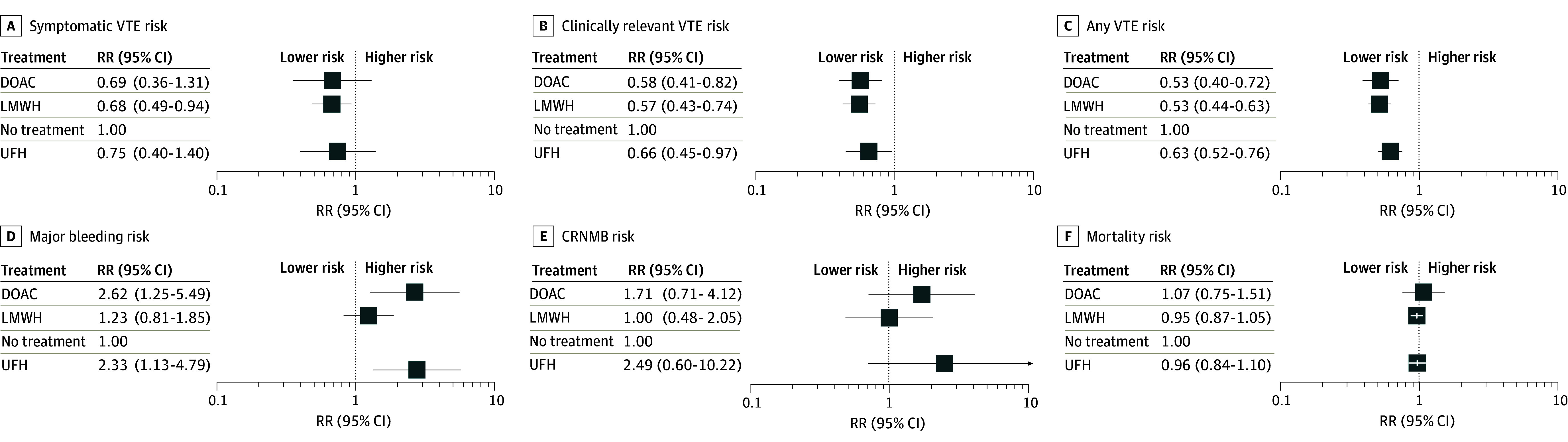
Forest Plots of Network Estimates of the Main Outcomes Error bars represent 95% CIs. CRNMB indicates clinically relevant nonmajor bleeding; DOAC, direct oral anticoagulants; LMWH; low-molecular-weight heparin; RR, relative risk; and UFH, unfractionated heparin.

Compared with no treatment, LMWH was associated with reduced symptomatic VTE risk (RR, 0.68; 95% CI, 0.49-0.94) (Figures 1 and 2). Point estimates for DOACs (RR, 0.69; 95% CI, 0.36-1.31) and UFH (RR, 0.75; 95% CI, 0.40-1.40) were less than 1, although the results were not statistically significant. There was no significant difference in risk between thromboprophylaxis regimens (Table 2 and Figure 1).

**Table 2.** Direct and Network Estimates for Symptomatic VTE, Clinically Relevant VTE, and Major Bleeding

Treatment	Comparative group	No. of studies	No. of participants	*I*^2^, %	RR (95%CI)			Inconsistency or overall heterogeneity *P* value^a^	Direct proportion, %^b^
					Direct estimate	Indirect estimate	Overall estimate		
**Symptomatic VTE**									
DOAC	LMWH	2^36,37^	14 526	1.6	1.01 (0.58-1.76)	NA	1.01 (0.58-1.76)	NA	100
DOAC	No treatment	0	NA	NA	NA	0.69 (0.36-1.31)	0.69 (0.36-1.31)		0
DOAC	UFH	0	NA	NA	NA	0.91 (0.42-1.98)	0.91 (0.42-1.98)		0
LMWH	No treatment	6^4,5,6,7,32,34^	15 710	0.0	0.68 (0.49-0.94)	NA	0.68 (0.49-0.94)		100
LMWH	UFH	6^25,26,30,33,34,35^	4315	0.0	0.91 (0.53-1.55)	NA	0.91 (0.53-1.55)		100
UFH	No treatment	0	NA	NA	NA	0.75 (0.40-1.40)	0.75 (0.40-1.40)		0
**Clinically relevant VTE**									
DOAC	LMWH	2^36,37^	11 476	0.0	1.02 (0.81-1.28)	NA	1.02 (0.81-1.28)	Inconsistency: .40; heterogeneity: .99	100
DOAC	No treatment	0	NA	NA	NA	0.58 (0.41-0.82)	0.58 (0.41-0.82)		0
DOAC	UFH	0	NA	NA	NA	0.87 (0.61-1.24)	0.87 (0.61-1.24)		0
LMWH	No treatment	4^4,5,30,32^	6868	0.0	0.58 (0.44-0.76)	0.26 (0.04-1.59)	0.57 (0.43-0.74)		97.8
LMWH	UFH	7^4,6,26,29,30,34,35^	5768	0.0	0.84 (0.63-1.11)	1.83 (0.31-10.98)	0.85 (0.65-1.13)		97.6
UFH	No treatment	2^3,24^	189	0.0	0.32 (0.05-1.85)	0.69 (0.47-1.02)	0.66 (0.45-0.97)		4.6
**Major bleeding**									
LMWH	DOAC	2^36,37^	14 389	0.0	0.47 (0.25 0.86)	NA	0.47 (0.25-0.86)	NA	100
DOAC	No treatment	0	NA	NA	NA	2.62 (1.25-5.49)	2.62 (1.25-5.49)		0
DOAC	UFH	0	NA	NA	NA	1.13 (0.48-2.64)	1.13 (0.48-2.64)		0
LMWH	No treatment	7^4,5,6,7,10,31,32^	18 844	0.0	1.23 (0.81-1.85)	NA	1.23 (0.81-1.85)		100
LMWH	UFH	9^25,26,28,29,30,33,34,35,38^	7358	0.0	0.53 (0.29-0.95)	NA	0.53 (0.29-0.95)		100
UFH	No treatment	0	NA	NA	NA	2.33 (1.13-4.79)	2.33 (1.13-4.79)		0

Abbreviations: DOAC, direct oral anticoagulant; LMWH, low-molecular-weight heparin; NA, not available; RR, relative risk; UFH, unfractionated heparin; VTE, venous thromboembolism.

^a^ Inconsistency between direct and indirect evidence.

^b^ Proportion of evidence from direct comparison (%).

In a sensitivity analysis, we applied the conversion rate (from screened asymptomatic DVT to symptomatic VTE) for 5 trials^4,5,35,36,37^ (eTable 6 in Supplement 1). The RR estimates were 0.63 (95% CI, 0.48-0.83) for LMWH and 0.66 (95% CI, 0.44-0.99) for DOACs, compared with no treatment. Estimates using alternative conversion rates were similar overall (eTable 7 in Supplement 1).

#### Clinically Relevant VTE

Fifteen studies (involving 24 301 patients) reported on clinically relevant VTE.^3,4,5,10,24,25,26,29,30,32,33,34,35,36,37^ The pooled 90-day risk of clinically relevant VTE was 4.2% (95% CI, 3.3%-5.3%) in the no-treatment group.

LMWH (RR, 0.57; 95% CI, 0.43-0.74), DOACs (RR, 0.58; 95% CI, 0.41-0.82), and UFH (RR, 0.66; 95% CI, 0.45-0.97) were associated with decreased risk of clinically relevant VTE, compared with no treatment. No significant differences in risk between active treatment arms were observed (Table 2 and Figure 2).

#### Any VTE

Twenty studies (involving 37 606 patients) reported on any VTE.^2,3,4,5,6,10,24,25,26,27,28,29,30,32,33,34,35,36,37,38^ The pooled 90-day risk of any VTE was 7.7% (95% CI, 4.3%-13.3%) in the no-treatment group.

LMWH (RR, 0.53; 95% CI, 0.44-0.63), DOACs (RR, 0.53; 95% CI, 0.40-0.72), and UFH (RR, 0.63; 95% CI, 0.52-0.76) were associated with reduced risk of any VTE, compared with no treatment. In comparisons between active treatment arms, LMWH was superior to UFH (RR, 0.84; 95% CI, 0.71-0.99). No significant differences in risk were observed between other treatment arms (eTable 8 in Supplement 1; Figure 2).

#### Major Bleeding

Eighteen studies (involving 40 591 patients) reported on major bleeding.^4,5,6,7,10,25,26,28,29,30,31,32,33,34,35,36,37,38^ The pooled 90-day risk of major bleeding was 0.5% (95% CI, 0.2%-1.2%) in the no-treatment group.

In the network meta-analysis, DOACs (RR, 2.62; 95% CI, 1.25-5.49) and UFH (RR, 2.33; 95% CI, 1.13-4.79) were associated with an increased risk of major bleeding compared with no treatment, but LMWH did not have such association (RR, 1.23; 95% CI, 0.81-1.85). In direct comparison, LMWH was associated with a lower risk of major bleeding compared with UFH (RR, 0.53; 95% CI, 0.29-0.95) and DOACs (RR, 0.47; 95% CI, 0.25-0.86) (Table 2 and Figure 2).

#### Clinically Relevant Nonmajor Bleeding

Six studies (involving 25 986 patients) reported on CRNMB.^7,10,30,33,36,37^ Compared with no treatment, LMWH was not associated with an increased risk of CRNMB (RR, 1.00; 95% CI, 0.48-2.05). DOACs and UFH had both greater risks of CRNMB, compared with LMWH or no treatment, although these latter comparisons were not statistically significant (eTable 8 in Supplement 1).

#### Mortality

No treatment regimen was associated with reduced risk for all-cause mortality (eTable 8 in Supplement 1; Figure 2). The point estimates showed close to no association.

### Sensitivity Analyses

**Figure 3.**
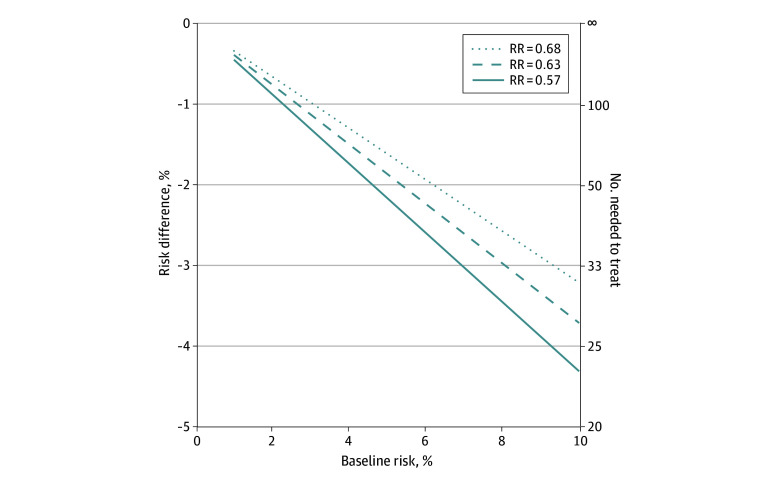
Line Graph of Expected Absolute Risk Reduction for Low-Molecular-Weight Heparin From Baseline Risk The relative risk (RR) was 0.68 (95% CI, 0.49-0.94) for symptomatic venous thromboembolism (VTE), 0.63 (95% CI, 0.48-0.83) for symptomatic VTE with the conversion of asymptomatic screened deep vein thrombosis, and 0.57 (95% CI, 0.43-0.74) for clinically relevant VTE.

The 28-day outcome analysis yielded similar RR estimates as that for the 90-day outcome (eTable 9 in Supplement 1). Exclusion of studies using ^125^I-labeled fibrinogen scan did not affect estimates and only decreased precision for the comparison of UFH to no treatment for the clinically relevant VTE (eTable 10 in Supplement 1).^2,3,26^ The leave-one-out meta-analysis for symptomatic VTE did not sensibly alter comparison estimates. To illustrate the importance of baseline VTE risks on absolute risk reductions, we computed absolute risk reductions for a range of baseline risks; the results of this analysis are provided in Figure 3.

### Sources of Heterogeneity and Publication Bias

A low level of heterogeneity (*I*^2^ < 25%) was observed for symptomatic VTE (*I*^2^ = 1.6%), clinically relevant VTE (*I*^2^ = 0.0%), and major bleeding (*I*^2^ = 0.0%) (Table 2). There were some differences in RR estimates between direct and indirect evidence for clinically relevant VTE (eg, LMWH vs no treatment: direct RR, 0.58 [95% CI, 0.44-0.76]; indirect RR, 0.26 [95% CI, 0.04-1.59]), although *P* values for comparisons were not statistically significant. The proportion of direct evidence in the network analysis varied, and several comparisons relied mainly or solely on indirect evidence (DOACs vs no treatment, DOACs vs UFH, and UFH vs no treatment). There was no statistically significant evidence of inconsistency (Table 2).

Visual inspection of the funnel plots for symptomatic VTE and major bleeding did not suggest publication bias, except possibly for the comparison between UFH and LMWH for major bleeding (eFigure 4 in Supplement 1). When applying the trim-and-fill method, the increased risk of major bleeding associated with UFH, compared with LMWH, was less marked (eFigure 5 in Supplement 1).

### Treatment Ranking

According to the network meta-analysis, LMWH was associated with the highest P-scores in effectiveness outcomes: 0.71 for symptomatic VTE, 0.81 for clinically relevant VTE, and 0.70 for mortality. The no-treatment arm was associated with the highest P-score for major bleeding (0.94) and second-highest P-score for CRNMB (0.76) (eFigure 3 in Supplement 1).

### GRADE Interpretation of the Findings

The GRADE interpretation of our findings for LMWH is reported in eFigure 6 in Supplement 1. The certainty of evidence was considered moderate for most outcomes (except mortality) due to the inclusion of open-label RCTs.^22^

## Discussion

In this network meta-analysis of 22 RCTs with a total of 43 840 medical inpatients, currently approved LMWH was significantly associated with reduced risk of clinically relevant VTE. The RR reduction for symptomatic VTE appeared similar for UFH and DOACs, although with less precision. There were differences in safety, with DOACs and UFH provoking twice as many major bleeding and CRNMB events, compared with LMWH. Accordingly, P-scores favored LMWH over other regimens for both effectiveness and safety outcomes. None of these treatments affected the all-cause mortality risk.

The present meta-analysis supports previous evidence. The 2007 meta-analysis by Dentali et al^39^ focused on symptomatic VTE and included 9 trials of medical inpatients. Compared with no treatment, a 53% to 57% risk reduction of PE and symptomatic DVT was associated with LMWH or UFH during the on-treatment period, without any statistically significant increase in major bleeding.^39^ Our meta-analysis involved more participants; stratified the results between LMWH, UFH, and DOACs; and used 90-day data instead of the on-treatment period only. The 2022 network meta-analysis by Eck et al^9^ evaluated thromboprophylaxis in 44 trials of inpatients with acute illness, critical illness, cardiologic disease, and stroke. Risk reductions of symptomatic VTE were observed for intermediate-dose LMWH, UFH, and DOACs compared with no treatment.^9^ Our study adds the important findings from the SYMPTOMS (Systematic Elderly Medical Patients Thromboprophylaxis: Efficacy on Symptomatic Outcomes) trial and presents results that are more generalizable to unselected acutely ill medical inpatients who carry distinct bleeding and thrombotic risks compared with selected populations in the ICU, with stroke, or with acute coronary syndrome. Furthermore, our study considered clinically relevant VTE by including asymptomatic proximal DVT. Proximal asymptomatic DVT is an important outcome, with a substantial risk of progression to symptomatic VTE and a poor outcome if left untreated.^13,15,40^ Finally, the present study included only currently approved medications, enhancing applicability.

The comparative effectiveness of LMWH for different VTE outcomes deserves attention. Our study suggests a 32%, 43%, and 47% RR reduction by LMWH for symptomatic VTE (RR, 0.68), clinically relevant VTE (RR, 0.57), and any VTE (RR, 0.53). When accounting for the bias induced by screened asymptomatic events leading to anticoagulation, the RR reduction for symptomatic VTE was 37% (RR, 0.63). Taken together, our data are compatible with an approximately 40% risk reduction of clinically relevant VTE. This rate is somewhat lower than for any VTE in our study and previous meta-analyses^41^ and suggests a possible overestimation of thromboprophylaxis benefits in nonblinded studies using systematic screening of asymptomatic VTE. Eck et al^9^ reported a pattern toward higher effectiveness and lower harm estimates in nonblinded RCTs compared with placebo-controlled studies.

Among currently approved regimens, LMWH was associated with the most favorable safety and effectiveness profile and the largest amount of evidence with direct comparisons to all other treatment arms. LMWH had the highest P-score for all effectiveness outcomes and the second-best P-score after no treatment for bleeding complications. Both UFH and DOACs were found to be associated with higher risk of major bleeding and CRNMB compared with LMWH, which had no strong safety signal. Based on these results, we would consider LMWH as the preferred option for medical thromboprophylaxis in acutely ill medical inpatients.

Our results have clinical implications. First, LMWH seemed to be the preferred option for medical thromboprophylaxis. Second, the limited magnitude of effectiveness (approximately 40%) for VTE outcomes highlights the need for risk stratification. From a patient and societal perspective, absolute risk reduction is most important. In the present meta-analysis dedicated to unselected medical inpatients, the pooled risk estimates for 90-day symptomatic VTE and clinically relevant VTE in the no-treatment group were 1.7% and 4.2%, respectively, translating into numbers needed to treat of about 200 to prevent 1 symptomatic VTE and 55 to prevent 1 clinically relevant VTE. Although LMWH appears safe for bleeding complications, the burden of injections and costs do not allow their universal use. Using specific risk assessment models at hospital admission remains valuable to stratify patients based on individual baseline VTE risks. This approach enables personalized thromboprophylaxis for those at higher baseline risk, with lower numbers needed to treat and improved cost-effectiveness.^42^ We illustrated the association between baseline risk and expected absolute benefit of thromboprophylaxis in Figure 3. Identifying a clear threshold to prescribe hospital thromboprophylaxis remains challenging, with local and patient preferences.^43^ However, a previous decision model suggested that patients with an absolute VTE risk of 1.0% or higher would benefit from in-hospital thromboprophylaxis.^44^

### Strengths and Limitations

Our study has several strengths. First, we conducted a thorough literature search to provide an exhaustive and contemporary summary of the best available evidence on the topic. Second, we selected RCTs dedicated to nonselected medical inpatients to improve applicability to this population. Third, we aimed to support clinical decisions by reporting on clinically relevant outcomes, applying our findings to different baseline risks, and evaluating treatment ranking for effectiveness and safety outcomes.

Fourth, this meta-analysis was the first, to our knowledge, to report both on symptomatic VTE and clinically relevant VTE and to apply a theoretical conversion of asymptomatic into symptomatic VTE. This approach takes into consideration pitfalls in pharmacologic thromboprophylaxis estimates, such as overestimation due to systematically detected asymptomatic events and underestimation due to selective therapeutic anticoagulation of asymptomatic events. Fifth, we provided estimates for both direct and network comparisons to consider direct and indirect evidence, and we contributed to explaining contrasted data observed in previously published evidence.

Our study also has limitations. First, systematic reviews and meta-analyses rely on the quality of included studies. Although most included RCTs were considered at low risk of bias, several studies were not blinded. Moreover, the number of included studies comparing DOACs with LMWH and the number of events in some comparison arms were small, which limits the precision of RR estimates and precludes analyzing additional sources of heterogeneity. Moreover, some RR estimates were based solely on indirect evidence, limiting our exploration of inconsistency and downgrading the certainty of evidence for comparisons informed mainly by indirect evidence. Second, we compared different classes of anticoagulants, but the number of available studies was insufficient to compare individual molecules within a given class. Nevertheless, we pooled the results of these different molecules and regimens without observing a large amount of heterogeneity.

Third, network meta-analyses depend on the comparative groups selected in clinical trials. Some RR estimates were based solely on indirect evidence, which assumed transitivity. While our study had limited assessment of transitivity, the absence of reported modifiers of effectiveness and safety outcomes in large RCTs^4^ suggests a low likelihood of transitivity issues. Fourth, we pooled no treatment and placebo as the same comparator, but these 2 categories might be considered as a potential source of heterogeneity. Eck et al^9^ reported a pattern toward larger benefits and lower harms in studies using no intervention, rather than placebo, as the reference. Finally, the duration of hospitalization exceeded 6 days in most of the included studies, which limits generalizability to shorter durations of hospital stay.

## Conclusions

In this systematic review and network meta-analysis, pharmacologic thromboprophylaxis was associated with effective prevention of symptomatic VTE and clinically relevant VTE in acutely ill medical inpatients. LMWHs were associated with a lower risk of major bleeding compared with DOACs and UFH. A thorough selection of patients is critical given the overall low absolute risk of VTE.

## Notes

Supplement 1. **eTable 1.** Detailed Methods **eTable 2.** Detailed Search Strategy **eTable 3.** Theoretical Conversion Rate of Asymptomatic Into Potentially Symptomatic Events **eTable 4.** Characteristics of Included Studies **eTable 5.** Risk of Bias of Included Studies **eTable 6.** Sensitivity Analysis Using a Theoretical Conversion of Asymptomatic Events **eTable 7.** Sensitivity Analysis Using Alternative Conversion Rates of Asymptomatic Events **eTable 8.** Direct and Network Estimates of Treatment Effect for Any VTE, Clinically Relevant Non-Major Bleeding and All-Cause Mortality **eTable 9.** Sensitivity Analysis for 28 Days-Outcomes **eTable 10.** Sensitivity Analysis Excluding Studies Using ^125^I-labeled Fibrinogen Scan **eFigure 1.** Study Flowchart **eFigure 2.** Diagram of the Network Meta-Analysis **eFigure 3.** P-Scores **eFigure 4.** Publication Bias (Funnel Plots) **eFigure 5.** Publication Bias Trim and Fill **eFigure 6.** GRADE Certainty of Evidence

Supplement 2.
Data Sharing Statement
