# Error in Byline

**DOI:** 10.1001/jamanetworkopen.2026.24522

**Published:** 2026-06-29

In the Original Investigation titled “Pharmacologic Thromboprophylaxis in Medical Inpatients: A Systematic Review and Network Meta-Analysis,” published May 15, 2026, Laura Girardi’s academic degree was incorrectly listed as PhD instead of MD in the author byline. This article has been corrected.^1^
