# Supplementary material for: Pharmacologic Thromboprophylaxis in Medical Inpatients: A Systematic Review and Network Meta-Analysis
Source: JAMA Netw Open. 2026 May 15;9(5):e2611449. doi: 10.1001/jamanetworkopen.2026.11449 (PMC13179560; doi:10.1001/jamanetworkopen.2026.11449)
Supplement: Supplement 1. — eTable 1. Detailed Methods eTable 2. Detailed Search Strategy eTable 3. Theoretical Conversion Rate of Asymptomatic Into Potentially Symptomatic Events eTable 4. Characteristics of Included Studies eTable 5. Risk of Bias of Included Studies eTable 6. Sensitivity Analysis Using a Theoretical Conversion of Asymptomatic Events eTable 7. Sensitivity Analysis Using Alternative Conversion Rates of Asymptomatic Events eTable 8. Direct and Network Estimates of Treatment Effect for Any VTE, Clinically Relevant Non-Major Bleeding and All-Cause Mortality eTable 9. Sensitivity Analysis for 28 Days-Outcomes eTable 10. Sensitivity Analysis Excluding Studies Using 125I-labeled Fibrinogen Scan eFigure 1. Study Flowchart eFigure 2. Diagram of the Network Meta-Analysis eFigure 3. P-Scores eFigure 4. Publication Bias (Funnel Plots) eFigure 5. Publication Bias Trim and Fill eFigure 6. GRADE Certainty of Evidence [file jamanetwopen-e2611449-s001.pdf]

Supplemental Online Content

Marti C, Righini M, Le Gal G, et al. Pharmacologic thromboprophylaxis in medical inpatients: a systematic review and network meta-analysis. *JAMA Netw Open*. 2026;9(5):e2611449 doi:10.1001/jamanetworkopen.2026.11449

eTable 1. Detailed Methods

eTable 2. Detailed Search Strategy

eTable 3. Theoretical Conversion Rate of Asymptomatic Into Potentially Symptomatic Events

eTable 4. Characteristics of Included Studies

eTable 5. Risk of Bias of Included Studies

eTable 6. Sensitivity Analysis Using a Theoretical Conversion of Asymptomatic Events

eTable 7. Sensitivity Analysis Using Alternative Conversion Rates of Asymptomatic Events

eTable 8. Direct and Network Estimates of Treatment Effect for Any VTE, Clinically Relevant Non-Major Bleeding and All-Cause Mortality

eTable 9. Sensitivity Analysis for 28 Days-Outcomes

eTable 10. Sensitivity Analysis Excluding Studies Using ^{125}I-labeled Fibrinogen Scan

eFigure 1. Study Flowchart

eFigure 2. Diagram of the Network Meta-Analysis

eFigure 3. P-Scores

eFigure 4. Publication Bias (Funnel Plots)

eFigure 5. Publication Bias Trim and Fill

eFigure 6. GRADE Certainty of Evidence

This supplemental material has been provided by the authors to give readers additional information about their work.

eTable 1: Detailed methods

**Eligibility criteria**<br>We included studies evaluating currently approved regimens for in-hospital TPX, divided into three groups: enoxaparin 40-60mg once daily (od.), nadroparin 2850-6000IU od., dalteparin 5000IU od., tinzaparin 4500-7000IU od., fondaparinux 2.5mg od. (grouped as LMWHs/Pentasaccharides); rivaroxaban 10mg od., apixaban 2.5mg twice daily (bid.), dabigatran 220mg od. (grouped as DOACs); UFH 10000-15000IU in 2 or 3 daily injections. The no treatment arm included placebo or no treatment depending on the design of included studies and was reported as the “no treatment” arm. We included studies evaluating in-hospital treatment and post-discharge treatment. For these studies, we extracted outcomes only for the in-hospital period. We excluded studies using non-standard prophylactic doses (e.g. enoxaparin 20mg), or commercially unavailable drugs. As such, we excluded trials evaluating betrixaban because of its withdrawal in 2020.
**Study Selection**<br>Non-relevant studies were excluded based on title and abstract. For potentially relevant studies, full text was obtained, and the investigators independently assessed study eligibility. Data on study design, patient characteristics and outcomes were extracted independently. Disagreements about study inclusion or data extraction were resolved by consensus or by discussion with a third author (JLR). We contacted ten corresponding authors by email about queries for missing data or design questions.
**Data extraction**<br>Bleeding events: We anticipated that most studies would use the International Society of Thrombosis and Haemostasis definitions for bleeding events, namely a decrease in hemoglobin level of ≥2g/dl, transfusion of ≥2 units of packed red blood cells, bleeding that occurs in a critical site or fatal bleeding, for major bleeding[1 2]; and overt bleeding that does not meet the criteria for major bleeding but is associated with medical intervention, unscheduled contact with a physician, interruption or discontinuation of study drug, or discomfort or impairment of activities of daily living, for CRNMB [1].
**Quality assessment**<br>We assessed the risk of bias (low, high or unknown) in the following domains: selection bias (random sequence generation, allocation concealment), performance bias (blinding of participants and personnel), detection bias (blinding of outcome assessment), attrition bias (incomplete outcome data), reporting bias (selective reporting), and other biases. For the

attrition bias, we assigned a high-risk for studies with ≥10% of incomplete data for the primary outcome, among all randomized participants. Two investigators assessed study quality independently. Disagreements were resolved by consensus or by a third reviewer if no consensus was found.

1. Kaatz S, Ahmad D, Spyropoulos AC, Schulman S, Subcommittee on Control of A. Definition of clinically relevant non-major bleeding in studies of anticoagulants in atrial fibrillation and venous thromboembolic disease in non-surgical patients: communication from the SSC of the ISTH. *J Thromb Haemost.* 2015;13(11):2119-2126.
2. Schulman S, Kearon C, Subcommittee on Control of Anticoagulation of the S, Standardization Committee of the International Society on T, Haemostasis. Definition of major bleeding in clinical investigations of antihemostatic medicinal products in non-surgical patients. *J Thromb Haemost.* 2005;3(4):692-694.

© 2026 Marti C et al. *JAMA Network Open*

eTable 2: Detailed Search Strategy[a]

Database	Search query
Pubmed	((venous thrombosis[MeSH Terms]) OR (Thromboembolism[MeSH Terms]) OR (venous thromboembolism[MeSH Terms]) OR (pulmonary embolism[MeSH Terms])) AND ((heparin, low molecular weight[MeSH Terms]) OR (lmwh[MeSH Terms]) OR (low molecular weight heparin[MeSH Terms]) OR (heparin, unfractionated[MeSH Terms]) OR (unfractionated heparin[MeSH Terms]) OR (fondaparinux[MeSH Terms]) OR (nadroparin[MeSH Terms]) OR (enoxaparin[MeSH Terms]) OR (tinzaparin[MeSH Terms]) OR (dalteparin[MeSH Terms]) OR (apixaban) OR (rivaroxaban[MeSH Terms]) OR (Dabigatran [MeSH Terms])) AND (prevention and control [MeSH Subheading])<br>With filter « randomized controlled trial »
Embase	(fondaparinux OR 'heparin'/exp OR 'low molecular weight heparin'/exp OR 'nadroparin'/exp OR 'enoxaparin'/exp OR 'tinzaparin'/exp OR 'dalteparin'/exp OR 'apixaban'/exp OR 'rivaroxaban'/exp OR 'dabigatran'/exp) AND ('lung embolism'/exp OR 'vein thrombosis'/exp OR 'venous thromboembolism'/exp) AND 'randomized controlled trial'/exp AND ([adult]/lim OR [aged]/lim) AND [humans]/lim AND [clinical study]/lim AND [embase]/lim AND prevention
Web of Science	TS=((venous thrombosis) OR (Thromboembolism) OR (venous thromboembolism) OR (pulmonary embolism)) AND TS=((lmwh) OR (low molecular weight heparin) OR (unfractionated heparin) OR (fondaparinux) OR (nadroparin) OR (enoxaparin) OR (tinzaparin) OR (dalteparin) OR (apixaban) OR (rivaroxaban) OR (Dabigatran)) AND ALL=((randomized clinical trial) OR (placebo-controlled trial)) AND ALL=(hospital prevention) NOT DT=(Review)
Cochrane register of clinical trials	#1 MeSH descriptor: [Venous Thromboembolism] explode all trees and with qualifier(s): [prevention & control - PC]<br>#2 MeSH descriptor: [Venous Thrombosis] explode all trees and with qualifier(s): [prevention & control - PC]<br>#3 MeSH descriptor: [Heparin] explode all trees<br>#4 MeSH descriptor: [Heparin, Low-Molecular-Weight] explode all trees<br>#5 MeSH descriptor: [Fondaparinux] explode all trees<br>#6 MeSH descriptor: [Nadroparin] explode all trees<br>#7 MeSH descriptor: [Enoxaparin] explode all trees<br>#8 MeSH descriptor: [Tinzaparin] explode all trees<br>#9 MeSH descriptor: [Dalteparin] explode all trees<br>#10 apixaban<br>#11 MeSH descriptor: [Rivaroxaban] explode all trees

© 2026 Marti C et al. *JAMA Network Open*

	#12	MeSH descriptor: [Dabigatran] explode all trees
	#13	#1 or #2
	#14	#3 or #4 or #5 or #6 or #7 or #8 #9 or #10 or # 11 or #12
	#15	#13 and #14
	#16	filter clinical trial

[a]No language restriction

© 2026 Marti C et al. *JAMA Network Open*

eTable 3: Theoretical conversion rate of asymptomatic into potentially symptomatic events

In case of asymptomatic distal DVT, if not treated:<br>- The risk of *pulmonary embolism* is 2.4% (95%CI 1.5-3.6), based on a meta-analysis showing 22 PE in 918 patients with isolated distal DVT without anticoagulation.[1] We assume that these events are symptomatic.<br>- The risk of proximal extension (proximal DVT) is 5.4%, based on the CACTUS trial.[2] The proportion of *symptomatic* (vs. asymptomatic) proximal DVT among proximal extensions is 10% (9/89), based on the proportions found in the PREVENT trial with systematic compression ultrasound at day 21.[3] This means that the risks of a *proximal symptomatic DVT* in case of an untreated asymptomatic distal DVT is assumed to be 0.5%.<br>- The risk of an asymptomatic distal DVT becoming a *symptomatic distal DVT* but not extending to a proximal DVT is 5% (expert-based estimate).<br>Therefore, we assume that asymptomatic distal DVT would have led, if untreated, to symptomatic VTE in 7.9% of cases.
In case of asymptomatic proximal DVT, if not treated:<br>- The risk of *symptomatic PE* is thought to be 50%.[4]<br>- The risk of becoming a *symptomatic proximal DVT* is 10%.[3]<br>Therefore, we assume that asymptomatic proximal DVT would have led, if untreated, to symptomatic VTE in 60% of cases.

References:

1. Franco L, Giustozzi M, Agnelli G, Becattini C. Anticoagulation in patients with isolated distal deep vein thrombosis: a meta-analysis. *J Thromb Haemost*. 2017;15(6):1142-1154.
2. Righini M, Galanaud JP, Guenneguez H, et al. Anticoagulant therapy for symptomatic calf deep vein thrombosis (CACTUS): a randomised, double-blind, placebo-controlled trial. *Lancet Haematol*. 2016;3(12):e556-e562.
3. Leizorovicz A, Cohen AT, Turpie AG, et al. Randomized, placebo-controlled trial of dalteparin for the prevention of venous thromboembolism in acutely ill medical patients. *Circulation*. 2004;110(7):874-879.
4. Kearon C. Natural history of venous thromboembolism. *Circulation*. 2003;107(23 Suppl 1):I22-30.

© 2026 Marti C et al. *JAMA Network Open*

eTable 4: Characteristics of included studies

	Study dates	Study sites	Immobility criteria	Diagnosis of VTE	Definition of major bleeding	Age, years (mean or median)	Men (%)	Obesity (%)	History of VTE (%)	Cancer (%)	Immobility (%)	Chronic heart failure
Gallus, 1973	n/a	1 Canadian hospital	Not required	Proximal DVT – phlebography<br>Distal DVT – I125-fibrinogen scan (+/- phlebography)<br>PE – n/a	n/a	64	75.6	n/a	n/a	n/a	n/a	n/a
Belch, 1981	n/a	1 UK hospital	Immobility for ≤2days	DVT – 125I-fibrinogen, then confirmation by CUS	n/a	65.8	69	22	n/a	n/a	n/a	n/a
Ibarra-Perez, 1988	n/a	1 Mexican hospital	Anticipated bed rest ≥3days	DVT – screening, then confirmation by phlebography<br>PE – likely lung scintigraphy	n/a	n/a	n/a	n/a	n/a	n/a	100	12
Aquino, 1991	n/a	1 French hospital	Not required	DVT – Doppler US and confirmation by phlebography<br>PE – lung scintigraphy	Intracranial, retroperitoneal, exteriorized, or Hb drop >20g/L	84.1	15	n/a	13	10	n/a	23
Forette, 1995	n/a	35 French hospitals	Recently transitory reduced mobility	DVT – CUS, if equivocal phlebography<br>PE – not stated	Bleeding requiring treatment cessation	83.3	25.1	16.3	10.5	5.1	n/a	27.1
Gardlund, 1996	1988-1991	6 Swedisch hospitals	Immobility	Fatal PE – necropsy<br>Non-fatal VTE – unclear	Not reported	75	n/a	n/a	n/a	20.9	n/a	36.8
Lechler, 1996 (THE PRIME)	1991-1993	26 German/Austrian hospitals	Expected 50% immobility for 7 days	DVT - CUS then confirmed by phlebography	ISTH definition	74	37.8	30.7	6.9	13.8	100	35.1

© 2026 Marti C et al. *JAMA Network Open*

				PE – perfusion scan, angiography or autopsy								
Harenberg, 1996	n/a	10 German hospitals	Expected immobility for ≥10days	DVT – CUS +/- phlebography<br>PE – lung scintigraphy +/- pulmonary angiography	Hb drop >30g/L; cerebral, intestinal or retroperitoneal bleeding	70.5	45	31.6	7.2	n/a	100	44
Samama, 1999 (MEDENOX)	1996-1998	60 hospitals in 9 countries	Immobility for <3days prior to inclusion	DVT – venography or CUS<br>PE- high-probability lung scintigraphy, angiography, CTPA or autopsy	ISTH definition	73.6	49.2	19.4	9.3	13.7	n/a	33.5
Kleber, 2003 (THE PRINCE)	n/a	Multiple German hospitals	Immobility for >2/3 of the time, but not immobilized for >24h prior to inclusion	DVT – venography or autopsy<br>PE – VQ lung scan, pulmonary angiography or autopsy	ISTH definition	70.5	51.6	30.4	5.9	6.2	100	56
Leizorovicz, 2004 (PREVENT)	2001-2002	219 hospitals in 26 countries	Immobility not required and <4days of prior immobilization)	DVT – CUS or venography<br>PE – lung scintigraphy, CTPA, angiography	ISTH definition	68.5	48.1	30.4	3.9	5.2	n/a	50.8
Mahé, 2005	<1996	39 European hospitals	Immobility (unable to walk >10m alone)	VTE – unclear	Not defined	76.1	40.5	n/a	1.9	13.8	100	25.8
Léderlé, 2006	1999-2000	5 VA hospitals (USA)	Not required	DVT – positive diagnostic test<br>PE – high probability lung scintigraphy, pulmonary angiogram or autopsy	Bleeding Severity Index definition	71.7	98.5	n/a	4.7	4.7	n/a	24.6

© 2026 Marti C et al. *JAMA Network Open*

Cohen, 2006 (ARTEMIS)	2002-2003	35 hospitals in 8 countries	Expected immobility for ≥4days	DVT – venography<br>PE – high-probability lung scintigraphy, CTPA, pulmonary angiography or autopsy	ISTH definition	74.7	42.4	n/a	4.6	15.4	100	n/a
NCT00445328	~2007-2008	Multicenter in India	Not required	DVT – color doppler US<br>PE – lung scintigraphy, CTPA	ISTH definition	61.3	53.0	n/a	n/a	n/a	n/a	n/a
Riess, 2010 (CERTIFY)	2007-2009	172 (likely German) hospitals	Expected decreased mobility for ≥4days, but not >3days prior to inclusion	DVT – CUS<br>PE – lung scintigraphy, CTPA, pulmonary angiography or MRI	ISTH definition	78.8	40.9	n/a	n/a	n/a	100	n/a
Schellong, 2010 (CERTAIN)	2006-2007	Multiple German hospitals	Significant recently decreased mobility	DVT – CUS<br>PE – not defined	ISTH definition + warranting treatment cessation	70.6	47.5	27.6	4.5	8.6	100	33.5
Kakkar, 2011 (LIFENOX)	2008-2010	193 hospitals in Asia, Mexico and Tunisia	Not required	"Objective testing"	ISTH definition	65.5	62.7	10.5	0.6	4.4	n/a	n/a
Goldhaber, 2011 (ADOPT)	2007-2011	302 international hospitals	Moderate or severe mobility restriction	Objectively-diagnosed and adjudicated VTE	ISTH definition	66.8	49.1	44.4	4.1	9.7	27.2	47.0
Cohen, 2013 (MAGELLAN)	2007-2010	556 international hospitals	Complete immobility for ≥1days and expected decreased mobility for ≥4days	Objectively-diagnosed and adjudicated VTE	ISTH definition	71	54.2	15.2	4.7	17.0	100	34.5
Ischi, 2013	2008-2009	1 hospital in India	Expected non-ambulatory condition ≥3days if not in ICU	n/a	Fatal bleeding; requiring urgent blood transfusion.	54.4	60.5	n/a	n/a	n/a	n/a	n/a
Mottier, 2023 (SYMPTOMS)	2015-2020	47 Swiss and French hospitals	Not required	Objectively-driven and adjudicated	ISTH definition	81.5	40.5	n/a	7.1	6.3	65	6.3

© 2026 Marti C et al. *JAMA Network Open*

Legend: CTPA=computed tomography pulmonary angiography; CUS=compression ultrasound; DVT=deep vein thrombosis; ICU=intensive care unit; ISTH=International Society on Thrombosis and Haemostasis; MRI=magnetic resonance imaging; n/a=information not available; PE=pulmonary embolism; UK=United Kingdom; USA=United States of America; VA=Veteran Affairs; VTE=venous thrombo-embolism

© 2026 Marti C et al. *JAMA Network Open*

eTable 5: Risk of bias of included studies

	Random sequence generation	Allocation concealment	Blinding of participants and personnel	Blinding of outcome assessment	Incomplete outcome data	Selective reporting	Other bias
Gallus, 1973	+	+	+	+	−	+	+
Belch, 1981	?	?	−	+	+	+	−
Ibarra-Perez, 1988	?	?	−	?	−	?	−
Aquino , 1991	?	?	−	−	+	+	+
Forette, 1995	+	+	−	?	+	+	+
Gardlund, 1996	?	?	−	+	+	+	−

© 2026 Marti C et al. *JAMA Network Open*

Lechler, 1996 (THE PRIME)	?	?	+	+	+	+	+
Harenberg, 1996	−	?	+	+	−	+	−
Samama, 1999 (MEDENOX)	?	+	+	+	−	+	+
Kleber, 2003 (THE-PRINCE)	+	+	−	+	−	+	+
Leizorovicz, 2004 (PREVENT)	?	?	+	+	−	+	+
Mahé, 2005	+	+	+	+	+	+	−
Léderlé, 2006	+	+	+	?	+	+	+
Cohen, 2006 (ARTEMIS)	+	+	+	+	−	+	+
NCT00445328	?	?	−	?	−	+	−

© 2026 Marti C et al. *JAMA Network Open*

Riess, 2010 (CERTIFY)	+	+	+	+	−	+	+
Schellong), 2010 (CERTAIN)	+	+	−	+	−	+	−
Kakkar, 2011 (LIFENOX)	+	+	+	+	+	+	+
Goldhaber, 2011 (ADOPT)	+	+	+	+	−	+	+
Cohen) 2013 (MAGELLAN)	+	+	+	+	−	+	+
Ischi, 2013	+	?	+	?	+	+	+
Mottier, 2023 (SYMPTOMS)	+	+	+	+	+	+	+

© 2026 Marti C et al. *JAMA Network Open*

eTable 6: Sensitivity analysis using a theoretical conversion of asymptomatic events, for symptomatic venous thromboembolism

- Conversion of asymptomatic distal DVT to symptomatic VTE 7.9%
- Conversion of asymptomatic proximal VTE to symptomatic VTE 60%

Treatment	Comparative group	N studies	N Participants	I2	Direct estimate	Indirect estimate	Overall estimate	Direct proportion *
DOAC	LMWH	2	14526	0.0%	1.05 [0.78; 1.40]	.	1.05 [0.78; 1.40]	100
DOAC	No	0	.	.	.	0.66 [0.44; 0.99]	0.66 [0.44; 0.99]	0
DOAC	UFH	0	.	.	.	0.85 [0.48; 1.51]	0.85 [0.48; 1.51]	0
LMWH	No	6	15710	5.6%	0.63 [0.48; 0.83]	.	0.63 [0.48; 0.83]	100
LMWH	UFH	6	4315	0.0%	0.78 [0.44; 1.36]	.	0.81 [0.50; 1.33]	100
UFH	No	0	.	.	.	0.78 [0.44; 1.36]	0.78 [0.44; 1.36]	0

* Proportion of evidence from direct comparison (%)
Legend: DOAC=direct oral anticoagulant; LMWH=low-molecular-weight heparin; UFH=unfractionated heparin.

© 2026 Marti C et al. *JAMA Network Open*

eTable 7: Sensitivity analysis using alternative conversion rates of asymptomatic events, for symptomatic venous thromboembolism

Low case scenario

- Conversion of asymptomatic distal DVT to symptomatic VTE 3%
- Conversion of asymptomatic proximal VTE to symptomatic VTE 40%
-

Treatment	Comparative group	N studies	N Participants	I2	Direct estimate	Indirect estimate	Overall estimate	Direct proportion *
DOAC	LMWH	2	14526	0.0%	1.05 [0.75; 1.47]	.	1.05 [0.78; 1.40]	100
DOAC	No	0	.	.	.	0.68 [0.44; 1.06]	0.68 [0.44; 1.06]	0
DOAC	UFH	0	.	.	.	0.90 [0.49; 1.65]	0.90 [0.49; 1.65]	0
LMWH	No	6	15710	0.0%	0.65 [0.49; 0.87]	.	0.65 [0.49; 0.87]	100
LMWH	UFH	6	4315	0.0%	0.86 [0.52; 1.42]	.	0.86 [0.52; 1.42]	100
UFH	No	0	.	.	.	0.76 [0.42; 1.36]	0.76 [0.42; 1.36]	0

* Proportion of evidence from direct comparison (%)
Legend: DOAC=direct oral anticoagulant; LMWH=low-molecular-weight heparin; UFH=unfractionated heparin.

High case scenario

- Conversion of asymptomatic distal DVT to symptomatic VTE 15%
- Conversion of asymptomatic proximal VTE to symptomatic VTE 80%

Treatment	Comparative group	N studies	N Participants	I2	Direct estimate	Indirect estimate	Overall estimate	Direct proportion *
DOAC	LMWH	2	14526	0.0%	1.06 [0.81; 1.37]	.	1.06 [0.81; 1.37]	100

© 2026 Marti C et al. *JAMA Network Open*

DOAC	No	0	.	.	.	0.66 [0.46; 0.96]	0.66 [0.46; 0.96]	0
DOAC	UFH	0	.	.	.	0.84 [0.49; 1.46]	0.84 [0.49; 1.46]	0
LMWH	No	6	15710	0.0%	0.63 [0.48; 0.82]	.	0.63 [0.48; 0.82]	100
LMWH	UFH	6	4315	0.0%	0.80 [0.49; 1.29]	.	0.80 [0.49; 1.29]	100
UFH	No	0	.	.	.	0.79 [0.46; 1.37]	0.79 [0.46; 1.37]	0

* Proportion of evidence from direct comparison (%)
Legend: DOAC=direct oral anticoagulant; LMWH=low-molecular-weight heparin; UFH=unfractionated heparin.

© 2026 Marti C et al. *JAMA Network Open*

eTable 8: Direct and network estimates of treatment effect for any VTE, clinically relevant non-major bleeding and all-cause mortality

Treatment	Comparative group	N studies	N Participants	I2	Direct estimate	Indirect estimate	Overall estimate	Inconsistency P value*	Direct proportion **
ANY VTE									
DOAC	LMWH	2	11476	0.0%	1.02 [0.81; 1.28]	.	1.02 [0.81; 1.28]	0.7017	100
DOAC	No	0	.	.	.	0.53 [0.40; 0.72]	0.53 [0.40; 0.72]		0
DOAC	UFH	0	.	.	.	0.85 [0.64; 1.13]	0.85 [0.64; 1.13]		0
LMWH	No	5	7403	0.0%	0.54 [0.43; 0.68]	0.50 [0.36; 0.69]	0.53 [0.44; 0.63]		67.9
LMWH	UFH	9	6745	0.0%	0.82 [0.68; 1.00]	0.89 [0.63; 1.25]	0.84 [0.71; 0.99]		75.7
UFH	No	4	11982	53.8%	0.61 [0.47; 0.79]	0.66 [0.49; 0.88]	0.63 [0.52; 0.76]		56.4
CLINICALLY RELEVANT NON-MAJOR BLEEDING									
DOAC	LMWH	2	14389	77.1%	1.71 [1.04; 2.83]	.	1.71 [1.04; 2.83]	.	100
DOAC	No	0	.	.	.	1.71 [0.71; 4.12]	1.71 [0.71; 4.12]		0
DOAC	UFH	0	.	.	.	0.69 [0.18; 2.56]	0.69 [0.18; 2.56]		0
LMWH	No	2	10848	7.6%	1.00 [0.48; 2.05]	.	1.00 [0.48; 2.05]		100
LMWH	UFH	2	749	0.0%	0.40 [0.12; 1.35]	.	0.40 [0.12; 1.35]		100
UFH	No	0	.	.	.	2.49 [0.60; 10.22]	2.49 [0.60; 10.22]		0
ALL-CAUSE MORTALITY									
DOAC	LMWH	1	6591	.	1.12 [0.80; 1.56]	.	1.12 [0.80; 1.56]	0.5514	100
DOAC	No	0	.	.	.	1.07 [0.75; 1.51]	1.07 [0.75; 1.51]		0
DOAC	UFH	0	.	.	.	1.11 [0.77; 1.61]	1.11 [0.77; 1.61]		0
LMWH	No	7	18625	0.0%	0.96 [0.87; 1.07]	0.88 [0.66; 1.17]	0.95 [0.87; 1.05]		89
LMWH	UFH	7	6931	30.1%	0.94 [0.74; 1.19]	1.03 [0.85; 1.25]	1.00 [0.86; 1.15]		38.3
UFH	No	1	11693	.	0.94 [0.80; 1.10]	1.03 [0.79; 1.33]	0.96 [0.84; 1.10]		72.7

* P values for inconsistency between direct and indirect evidence
** Proportion of evidence from direct comparison (%)
Legend: DOAC=direct oral anticoagulant; LMWH=low-molecular-weight heparin; UFH=unfractionated heparin

© 2026 Marti C et al. *JAMA Network Open*

eTable 9: Sensitivity analysis 28 days-outcome

Treatment	Comparative group	N studies	N Participants	I2	Direct estimate	Indirect estimate	Overall estimate	Inconsistency P value*	Direct proportion **
SYMPTOMATIC VTE									
DOAC	LMWH	2	14526	1.6%	1.01 [0.58; 1.76]	.	1.01 [0.58; 1.76]	.	100
DOAC	No	0	.	.	.	0.65 [0.32; 1.33]	0.65 [0.32; 1.33]		0
DOAC	UFH	0	.	.	.	0.91 [0.37; 2.26]	0.91 [0.37; 2.26]		0
LMWH	No	4	6755	17.3%	0.65 [0.42; 1.00]	.	0.65 [0.42; 1.00]		100
LMWH	UFH	6	4216	0.0%	0.90 [0.44; 1.85]	.	0.90 [0.44; 1.85]		100
UFH	No	0	.	.	.	0.72 [0.31; 1.66]	0.72 [0.31; 1.66]		0
CLINICALLY RELEVANT VTE									
DOAC	LMWH	2	11476	0.0%	1.02 [0.81; 1.28]	.	1.02 [0.81; 1.28]	0.4015	100
DOAC	No	0	.	.	.	0.57 [0.39; 0.84]	0.57 [0.39; 0.84]		0
DOAC	UFH	0	.	.	.	0.87 [0.60; 1.27]	0.87 [0.60; 1.27]		0
LMWH	No	3	6176	4.2%	0.58 [0.43; 0.78]	0.26 [0.04; 1.59]	0.56 [0.42; 0.76]		97.2
LMWH	UFH	7	5465	0.0%	0.84 [0.62; 1.13]	1.83 [0.30; 11.03]	0.86 [0.64; 1.15]		97.3
UFH	No	2	189	0.0%	0.32 [0.05; 1.85]	0.69 [0.45; 1.06]	0.66 [0.43; 1.00]		5.5
ANY VTE									
DOAC	LMWH	2	11476	0.0%	1.02 [0.81; 1.28]	.	1.02 [0.81; 1.28]	0.8017	100
DOAC	No	0	.	.	.	0.53 [0.39; 0.71]	0.53 [0.39; 0.71]		0
DOAC	UFH	0	.	.	.	0.85 [0.63; 1.13]	0.85 [0.63; 1.13]		0
LMWH	No	4	6755	22.5%	0.53 [0.41; 0.68]	0.50 [0.36; 0.70]	0.52 [0.43; 0.63]		64
LMWH	UFH	9	6442	0.0%	0.82 [0.67; 1.01]	0.87 [0.61; 1.25]	0.84 [0.70; 1.00]		76.2
UFH	No	4	11982	53.8%	0.61 [0.47; 0.79]	0.64 [0.47; 0.88]	0.62 [0.51; 0.76]		59.8
MAJOR BLEEDING									
DOAC	LMWH	2	14389	0.0%	2.14 [1.16; 3.95]	.	2.14 [1.16; 3.95]		100
DOAC	No	0	.	.	.	2.73 [1.27; 5.87]	2.73 [1.27; 5.87]		0

DOAC	UFH	0	.	.	.	1.15 [0.49; 2.71]	1.15 [0.49; 2.71]	.	0
LMWH	No	6	18564	0.0%	1.28 [0.81; 2.02]	.	1.28 [0.81; 2.02]		100
LMWH	UFH	9	7358	0.0%	0.54 [0.30; 0.98]	.	0.54 [0.30; 0.98]		100
UFH	No	0	.	.	.	2.37 [1.12; 5.01]	2.37 [1.12; 5.01]		0
CLINICALLY RELEVANT NON-MAJOR BLEEDING									
DOAC	LMWH	2	14389	77.1%	1.72 [1.11; 2.67]	.	1.72 [1.11; 2.67]	.	100
DOAC	No	0	.	.	.	2.03 [0.88; 4.71]	2.03 [0.88; 4.71]		0
DOAC	UFH	0	.	.	.	0.69 [0.20; 2.41]	0.69 [0.20; 2.41]		0
LMWH	No	2	10848	0.0%	1.18 [0.58; 2.42]	.	1.18 [0.58; 2.42]		100
LMWH	UFH	2	749	0.0%	0.40 [0.12; 1.29]	.	0.40 [0.12; 1.29]		100
UFH	No	0	.	.	.	2.97 [0.75; 11.73]	2.97 [0.75; 11.73]		0
ALL-CAUSE MORTALITY									
DOAC	LMWH	1	6591	.	1.12 [0.80; 1.56]	.	1.12 [0.80; 1.56]	0.5858	100
DOAC	No	0	.	.	.	1.12 [0.78; 1.60]	1.12 [0.78; 1.60]		0
DOAC	UFH	0	.	.	.	1.18 [0.81; 1.72]	1.18 [0.81; 1.72]		0
LMWH	No	5	17725	0.0%	1.02 [0.88; 1.17]	0.91 [0.62; 1.33]	1.00 [0.87; 1.15]		87.2
LMWH	UFH	7	6807	29.7%	0.97 [0.68; 1.37]	1.09 [0.88; 1.34]	1.05 [0.88; 1.26]		26.6
UFH	No	1	11693	.	0.94 [0.80; 1.09]	1.05 [0.72; 1.53]	0.95 [0.83; 1.09]		86.2

* P values for inconsistency between direct and indirect evidence
** Proportion of evidence from direct comparison (%)
Legend: DOAC=direct oral anticoagulant; LMWH=low-molecular-weight heparin; UFH=unfractionated heparin; VTE=venous thrombo-embolism

© 2026 Marti C et al. *JAMA Network Open*

eTable 10: Sensitivity analysis excluding studies using ^{125}I-labelled fibrinogen scan, for the outcomes of clinically relevant venous thromboembolism (VTE) and any VTE

Arm 1	Arm 2	N studies	N participants	I2	Direct estimate	Indirect estimate	Overall estimate	Inconsistency P value*	Prop direct
Any venous thromboembolism									
DOAC	LMWH	2	11476	0.00%	1.02 [0.81; 1.28]	.	1.02 [0.81; 1.28]	0.8896	100
DOAC	No ttt	0	.	.	.	0.54 [0.40; 0.74]	0.54 [0.40; 0.74]		0
DOAC	UFH	0	.	.	.	0.83 [0.62; 1.11]	0.83 [0.62; 1.11]		0
LMWH	No ttt	4	6755	22.50%	0.53 [0.41; 0.68]	0.54 [0.39; 0.76]	0.53 [0.44; 0.65]		65
LMWH	UFH	9	6442	0.00%	0.82 [0.67; 1.01]	0.80 [0.56; 1.16]	0.82 [0.69; 0.98]		76.8
UFH	No ttt	1	11693	.	0.66 [0.50; 0.86]	0.64 [0.47; 0.88]	0.65 [0.53; 0.80]		58.2
Clinically relevant venous thromboembolism									
DOAC	LMWH	2	11476	0.00%	1.02 [0.81; 1.28]	.	1.02 [0.81; 1.28]	.	100
DOAC	No ttt	0	.	.	.	0.59 [0.40; 0.86]	0.59 [0.40; 0.86]		0
DOAC	UFH	0	.	.	.	0.85 [0.58; 1.24]	0.85 [0.58; 1.24]		0
LMWH	No ttt	3	6176	4.20%	0.58 [0.43; 0.78]	.	0.58 [0.43; 0.78]		100
LMWH	UFH	7	5465	0.00%	0.84 [0.62; 1.13]	.	0.84 [0.62; 1.13]		100
UFH	No ttt	0	.	.	.	0.69 [0.45; 1.06]	0.69 [0.45; 1.06]		0

© 2026 Marti C et al. *JAMA Network Open*

eFigure 1: Study Flow Chart

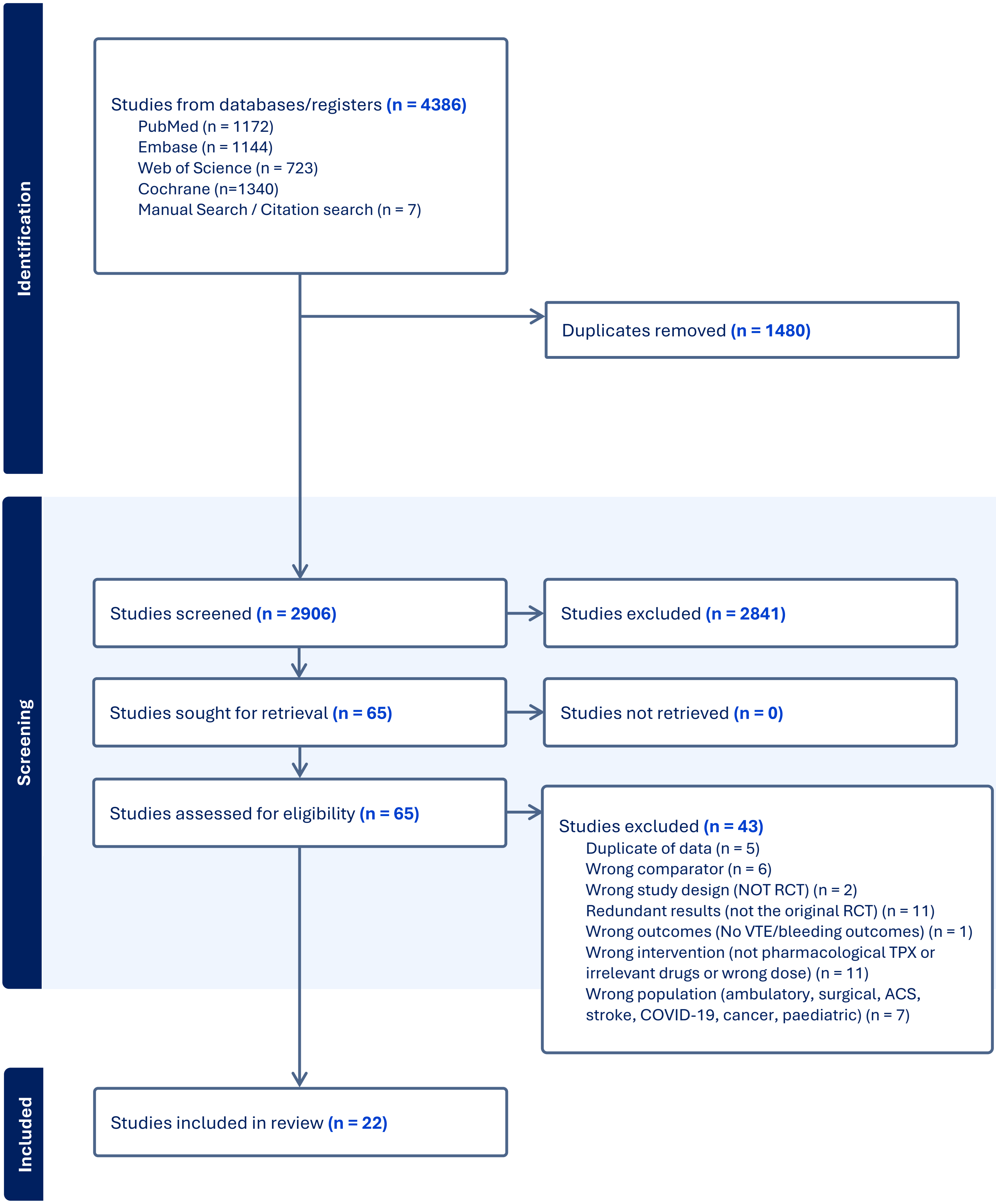

© 2026 Marti C et al. *JAMA Network Open*

eFigure 2: Diagram of the network meta-analysis

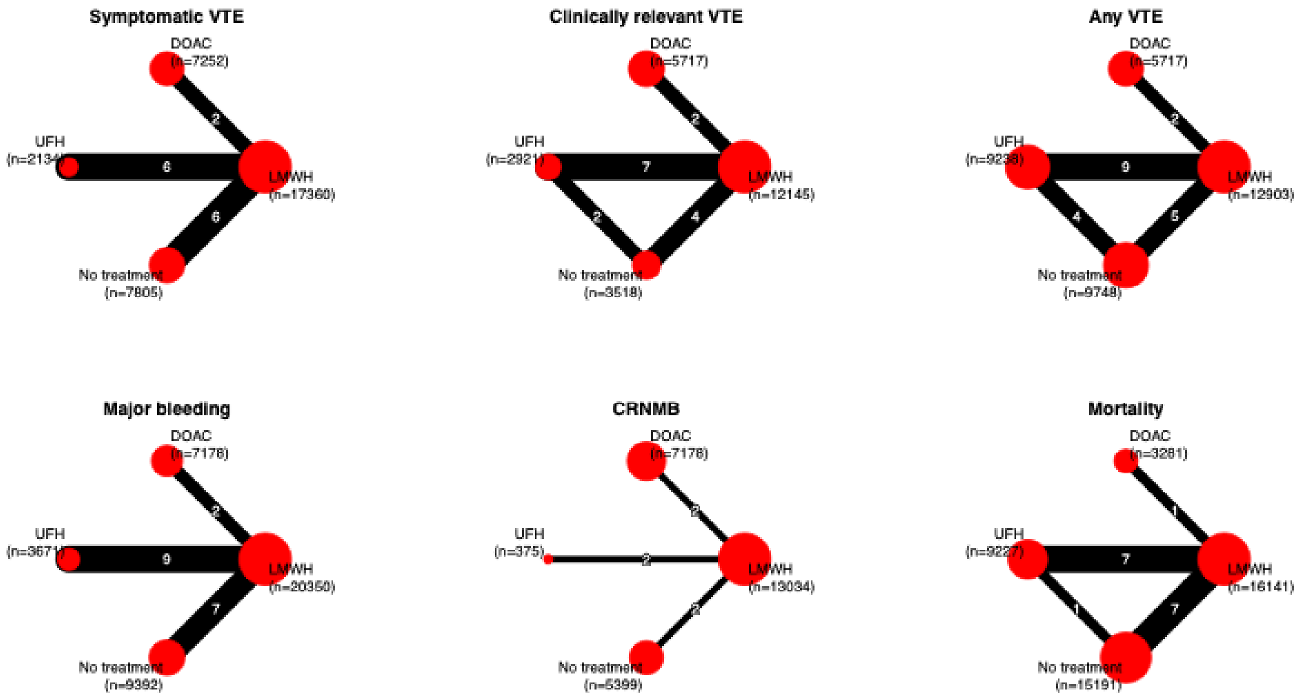

The size of the red circles is proportional to the number of patients (n= total number of patients) and the size of the black lines to the number of studies for each comparison (number of studies indicated in white on the line)

© 2026 Marti C et al. *JAMA Network Open*

eFigure 3: P-scores

The P-score can be interpreted as the mean extent of certainty that treatment is better than another treatment, averaged over all competing treatments.

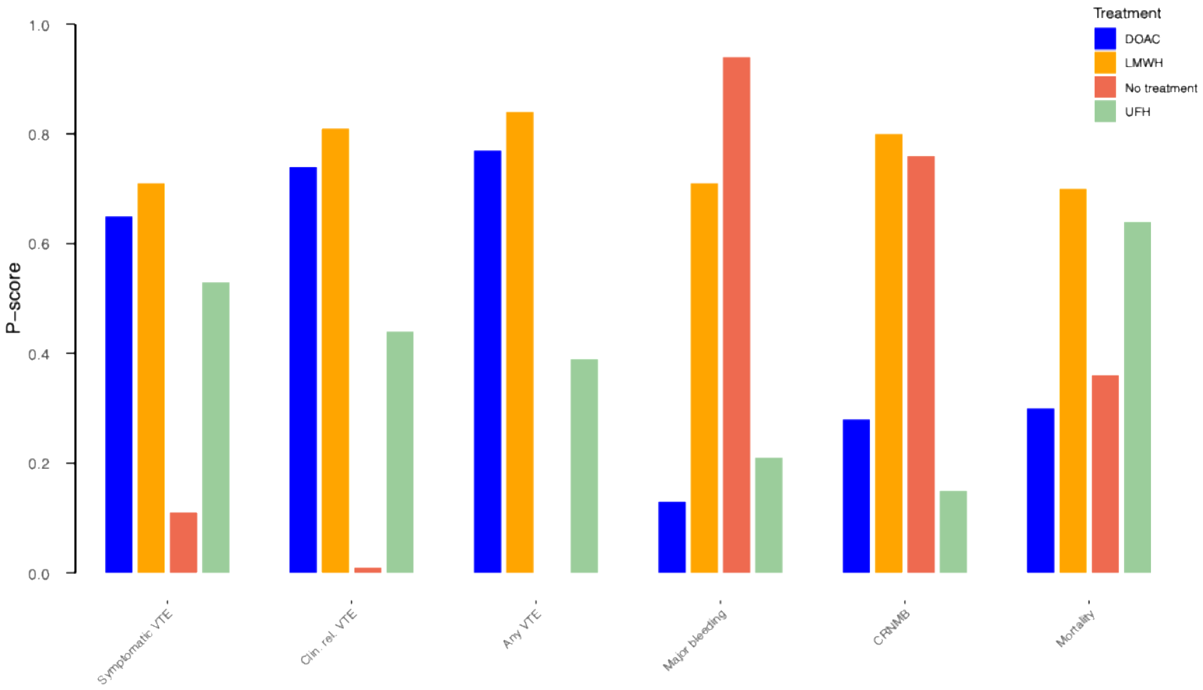

	Symptomatic VTE	Clinically relevant VTE	Major bleeding	Any VTE	CRNMB	Mortality
DOAC	0.65	0.74	0.13	0.77	0.28	0.30
LMWH	0.71	0.81	0.71	0.84	0.80	0.70
No TTT	0.11	0.01	0.94	0.00	0.76	0.36
UFH	0.53	0.44	0.21	0.39	0.15	0.64

Legend: CRNMB=clinically relevant non-major bleeding; DOAC=direct oral anticoagulant; LMWH=low-molecular-weight heparin; TTT=treatment; UFH=unfractionated heparin; VTE=venous thrombo-embolism.

© 2026 Marti C et al. *JAMA Network Open*

eFigure 4: Publication bias (Funnel Plots)

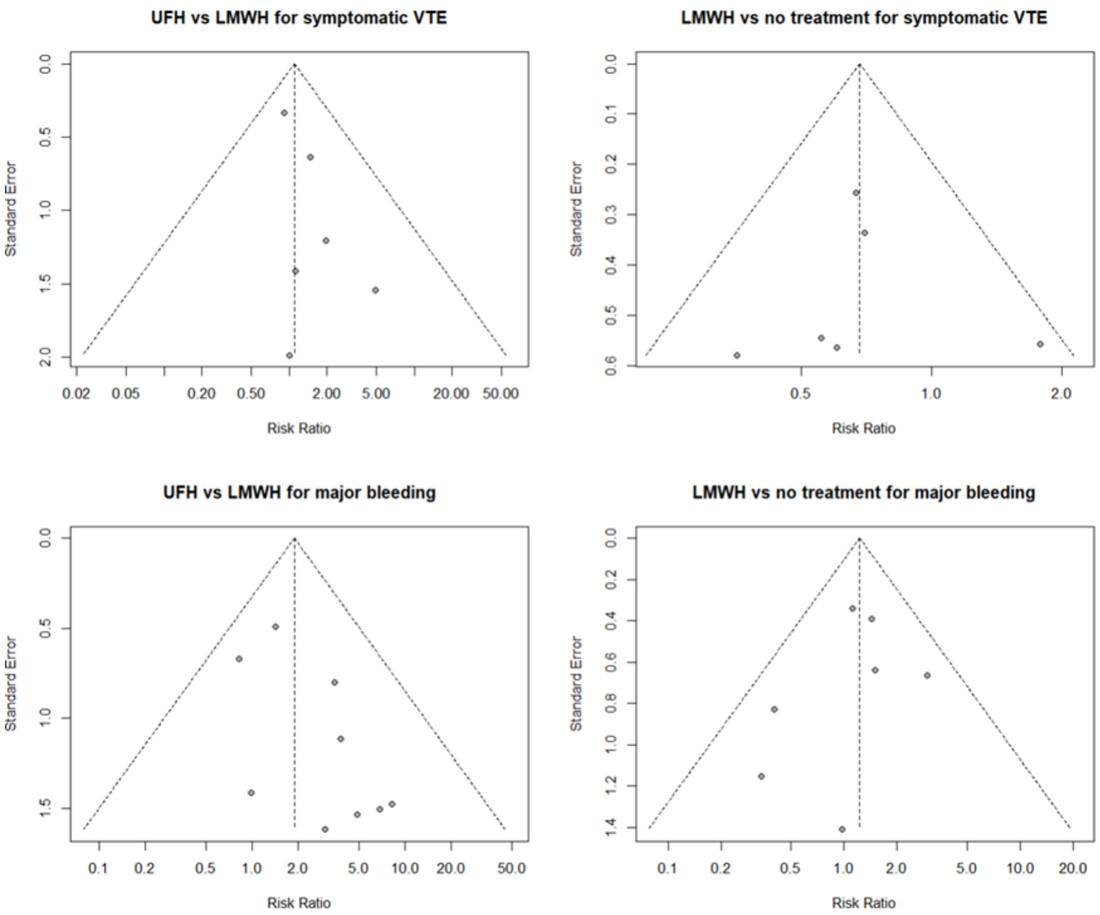

UFH: Unfractionated Heparin, LMWH: Low molecular weight heparin ; VTE: venous thromboembolism

© 2026 Marti C et al. *JAMA Network Open*

eFigure 5: Publication bias Trim and Fill

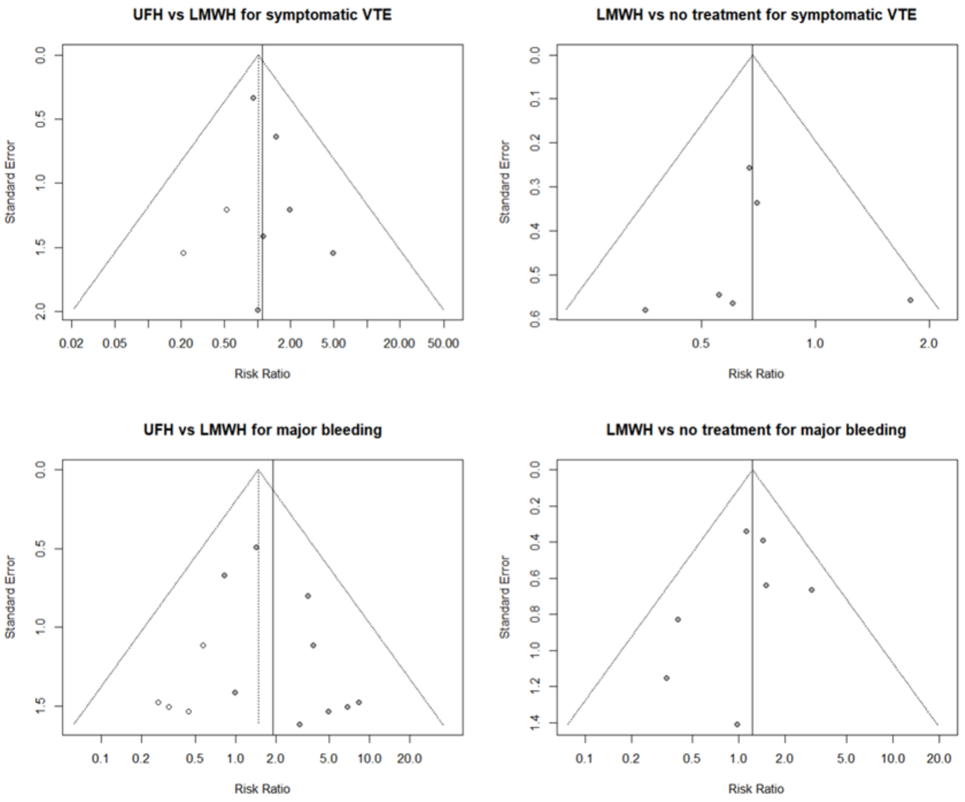

UFH: Unfractionated Heparin, LMWH: Low molecular weight heparin ; VTE: venous thromboembolism

Empty circles correspond to potentially missing studies. The vertical full line are the estimated pooled estimates. The vertical dashed line represents the pooled estimate with the addition of potentially unpublished studies (empty circles).

© 2026 Marti C et al. *JAMA Network Open*

eFigure 6: GRADE certainty of evidence for LMWH versus no treatment

Summary of Findings for LMWH Versus no treatment for medical inpatients

Outcome and follow-up	Patients (studies), N	Relative effect (95% CI)	Absolute effects (95% CI)			Certainty	What happens
			no treatment	LMWH	Difference		
			Pooled risk				
Symptomatic VTE	17360 (14 RCTs)	**RR = 0.68** (0.49 to 0.94)	17 per 1 000	**12 per 1 000** (8 to 16)	**5 fewer per 1 000** (from 9 fewer to 1 fewer)	⊕⊕⊕◯ Moderate[a]	LMWH likely results in a slight reduction in symptomatic VTE.
			Pooled risk				
Clinically relevant VTE	12145 (13 RCTs)	**RR = 0.57** (0.43 to 0.74)	42 per 1 000	**24 per 1 000** (18 to 31)	**18 fewer per 1 000** (from 24 fewer to 11 fewer)	⊕⊕⊕◯ Moderate[a]	LMWH likely results in a slight reduction in clinically relevant VTE.
			Pooled risk				
Any VTE	12903 (16 RCTs)	**RR = 0.53** (0.44 to 0.63)	77 per 1 000	**41 per 1 000** (34 to 49)	**36 fewer per 1 000** (from 43 fewer to 28 fewer)	⊕⊕⊕◯ Moderate[a]	LMWH probably results in a slight reduction in any VTE.
			Pooled risk				
Mortality	16141 (15 RCTs)	**RR = 0.90** (0.87 to 1.05)	78 per 1 000	**70 per 1 000** (68 to 82)	**8 fewer per 1 000** (from 10 fewer to 4 more)	⊕⊕◯◯ Low[a,b]	LMWH may result in little to no difference in mortality.
			Pooled risk				
Major bleeding	20350 (18 RCTs)	**RR = 1.23** (0.81 to 1.85)	5 per 1 000	**6 per 1 000** (4 to 9)	**1 more per 1 000** (from 1 fewer to 4 more)	⊕⊕⊕◯ Moderate[a,b]	LMWH likely results in little to no difference in major bleeding.

CI: confidence interval; **RR:** risk ratio

a. inclusion of open-label RCTs
b. conficdence intervall including clinically relevant difference

© 2026 Marti C et al. *JAMA Network Open*
