# Supplementary material for: Pharmacologic Thromboprophylaxis in Medical Inpatients: A Systematic Review and Network Meta-Analysis
Source: JAMA Netw Open. 2026 May 15;9(5):e2611449. doi: 10.1001/jamanetworkopen.2026.11449 (PMC13179560; doi:10.1001/jamanetworkopen.2026.11449)
Supplement: Supplement 2. — Data Sharing Statement [file jamanetwopen-e2611449-s002.pdf]

Data Sharing Statement

Marti. Pharmacologic Thromboprophylaxis in Medical Inpatients. *JAMA Netw Open*. Published May 15, 2026. doi:10.1001/jamanetworkopen.2026.11449

Data
Data available: No

Additional Information
Explanation for why data not available: No original individual data, study-level data will be shared upon reasonable request
